# A Cranberry Concentrate Decreases Adhesion and Invasion of *Escherichia coli* (AIEC) LF82 In Vitro

**DOI:** 10.3390/pathogens10091217

**Published:** 2021-09-18

**Authors:** Derek Zhang, Lynn Verstrepen, Jelle De Medts, Cindy Duysburgh, Pieter Van den Abbeele, Massimo Marzorati, Christina Khoo

**Affiliations:** 1 Ocean Spray Cranberries, Inc., Lakeville-Middleboro, MA 02349, USA; dzhang@oceanspray.com; 2 ProDigest BV, 9052 Ghent, Belgium; lynn.verstrepen@prodigest.eu (L.V.); jelle.demedts@prodigest.eu (J.D.M.); cindy.duysburgh@prodigest.eu (C.D.); massimo.marzorati@prodigest.eu (M.M.); 3 Cryptobiotix SA, 9052 Ghent, Belgium; pieter.vandenabbeele@cryptobiotix.eu; 4 Center of Microbial Ecology and Technology (CMET), Department of Biotechnology, Faculty of Bioscience Engineering, Ghent University, 9000 Ghent, Belgium

**Keywords:** AIEC, pathogen, cranberry, in vitro, adhesion, mucus, epithelium, prebiotic

## Abstract

While many beneficial host–microbiota interactions have been described, imbalanced microbiota in the gut is speculated to contribute to the progression and recurrence of chronic inflammatory diseases such as Crohn’s disease (CD). This in vitro study evaluated the impact of a cranberry concentrate Type M (CTM) on adherent-invasive *Escherichia coli* (AIEC) LF82, a pathobiont associated with CD. Different stages of pathogenic infection were investigated: (i) colonization of the mucus layer, and (ii) adhesion to and (iii) invasion of the epithelial cells. Following 48 h of fecal batch incubation, 0.5 and 1 mM of CTM significantly altered AIEC LF82 levels in a simulated mucus layer, resulting in a decrease of 50.5% in the untreated blank, down to 43.0% and 11.4%, respectively. At 1 mM of CTM, the significant decrease in the levels of AIEC LF82 coincided with a stimulation of the metabolic activity of the background microbiota. The increased levels of health-associated acetate (+7.9 mM) and propionate levels (+3.5 mM) suggested selective utilization of CTM by host microorganisms. Furthermore, 1 mM of both fermented and unfermented CTM decreased the adhesion and invasion of human-derived epithelial Caco-2 cells by AIEC LF82. Altogether, this exploratory in vitro study demonstrates the prebiotic potential of CTM and supports its antipathogenic effects through direct and/or indirect modulation of the gut microbiome.

## 1. Introduction

The human gut is inhabited by a vast number of bacteria that have a substantial impact on human health [1]. Although the majority of gut microbes are commensal or mutualistic, pathogenic bacteria such as *Salmonella*, *Shigella*, *Helicobacter*, *Vibrio*, *Campylobacter*, *Yersinia*, *Clostridia*, and *Listeria* can negatively affect human health via infectious diseases [2]. In contrast to pathogens that can often cause infectious disease in healthy hosts, pathobionts are defined as resident microbes that are innocuous to the host under normal conditions, yet associated with chronic inflammation [3]. Previous studies have shown correlations between the gut microbiome and chronic inflammatory diseases such as Crohn’s disease (CD) [4,5,6,7]. In view of CD, adherent-invasive *Escherichia coli* (AIEC) is a pathobiont of particular interest given its high prevalence in the inflamed ileal and colonic mucosa of CD patients [8]. It plays an important role in the induction and/or maintenance of intestinal inflammation, as reviewed by Chervy et al. [9]. Treatments should not only be developed to tackle the increasing incidence of CD (around 300 per 100,000 inhabitants in Europe/North America [10]), but also its social and economic burden (annual cost estimated to be around EUR 30 billion for Europe/North America in 2013 [11]).

Not everyone who ingests an infectious dose of a given pathogen will develop diseases [12]. In order to cause an infection, pathogens need to overcome several hurdles. First, pathogens need to be resistant to the actions of salivary enzymes and the acidic gastric environment [13]. Next, high concentrations of bile salts in the duodenum are toxic to certain bacterial groups [14,15]. Furthermore, the presence of a healthy, balanced host microbiota competes with pathogens for nutrients and space, and can produce metabolites that inhibit the growth and activities of pathogens [16]. If a pathogen surpasses these hurdles, it still needs to infect the host’s intestinal epithelial cells (IECs). This typically includes three steps: (i) adhesion to and penetration of the mucus layer that covers the surface of IECs, (ii) adhesion to IECs, and (iii) invasion of IECs [17]. AIEC is an interesting test strain for proof-of-concept studies, since it utilizes specialized strategies targeting each of these three steps. AIEC can penetrate the mucus layer by promoting mucin degradation with proteases [18]. Adhesion to IECs occurs via interactions between long polar fimbriae type 1 pili of AIEC and the carcinoembryonic antigen-related cell adhesion molecule 6 (CEACAM6) of the host [19,20]. AIEC utilizes bacterial effector delivery to invade host cells via outer membrane vesicles [21]. Different in vitro models have been applied to investigate AIEC colonization of the mucus layer [22] and its interaction with IECs [23]. Thus, these studies often focus on a single step of the infection process. However, it is important to investigate all three stages in order to obtain better insight into the antipathogenic effects.

The American cranberry (*Vaccinium macrocarpon*) is known to exert antipathogenic effects. In the course of urinary tract infections, uropathogenic *E. coli* adheres to uroepithelial cells via P-fimbriae [24]. Proanthocyanidins (PACs) (mostly A-type linkages) in cranberry products are capable of lowering this P-fimbriae-mediated adhesion [25,26]. Some lines of evidence also show that cranberries modulate microbiota in the gut [27]. In the study performed by Cai et al., dietary cranberry significantly suppressed colonic pro-inflammatory cytokines and decreased the abundance of potentially harmful bacteria, such as *Sutterella* and *Bilophila,* in dextran sodium sulfate treated mice. When dosed using an in vitro gut model, cranberries suppressed *Enterobacteriaceae* (to which *E. coli* belongs) and increased *Bacteroidaceae* [28]. Cranberry and its active ingredients, i.e., polyphenols such as anthocyanins, anthocyanidins, PACs, phenolics, and flavonols [29], are interesting candidates with which to develop strategies to combat pathogen-mediated infections.

This study aimed to investigate the antipathogenic effects of a cranberry concentrate Type M (CTM) against AIEC LF82 using an integrated in vitro approach to assess (i) adhesion to a simulated mucus layer in the presence of a simulated background microbiota, (ii) adhesion to human-derived epithelial cells (caco2-cells), and (iii) invasion of these epithelial cells.

## 2. Materials and Methods

### 2.1. Chemicals

All chemicals were obtained from Sigma-Aldrich (Bornem, Belgium) unless otherwise stated. The CTM was supplied by Ocean Spray Cranberries Inc. (Lakeville-Middleboro, MA, USA). The composition of CTM was characterized (Table 1) and CTM was tested at concentrations of 0.5–1 mM proanthocyanidin (PAC). Gu et al. quantified that cranberry juice contains 231 mg PAC/L or around 60 mg per serving [30]. Similar as how Deprez et al. estimated that an intake of 45 mg PAC would result in a minimum concentration of 0.3 mM in the intestine [31], intake of 1–2 servings of cranberry juice would thus result in a concentration in the range of 0.5–1 mM which was thus selected as concentration range for the current project.

### 2.2. Single Strain and Growth Conditions

The ampicillin/erythromycin-resistant AIEC LF82, isolated from a chronic ileal lesion of a CD patient [32], was used as the AIEC reference strain. AIEC was grown in a nutrient broth at 37 °C under aerobic conditions upon inoculating 1% from a frozen stock stored at −80 °C with 20% (*v*/*v*) of glycerol.

Two media were used during fecal batch incubations. In Test 1, the background medium consisted of 5.2 g/L of K_2_HPO_4_, 16.3 g/L of KH_2_PO_4_, 2.0 g/L of NaHCO_3_ (Chem-lab NV, Zedelgem, Belgium), 2.0 g/L of Yeast Extract (Oxoid, Aalst, Belgium), 2.0 g/L of pepton (Oxoid, Aalst, Belgium), 1.0 g/L of mucin (Carl Roth, Karlsruhe, Germany), 0.5 g/L of L-cystein, and 2.0 mL/L of Tween80. In Test 2, the concentrated background medium consisted of 7.6 g/L of K_2_HPO_4_, 23.9 g/L of KH_2_PO_4_, 2.9 g/L of NaHCO_3_ (Chem-lab NV, Zedelgem, Belgium), 2.9 g/L of Yeast Extract (Oxoid, Aalst, Belgium), 2.9 g/L of pepton (Oxoid, Aalst, Belgium), 1.5 g/L of mucin (Carl Roth, Karlsruhe, Germany), 0.7 g/L of L-cystein, and 2.9 mL/L of Tween80.

### 2.3. Test 1: Fecal Batch Incubation

**Figure 1:**
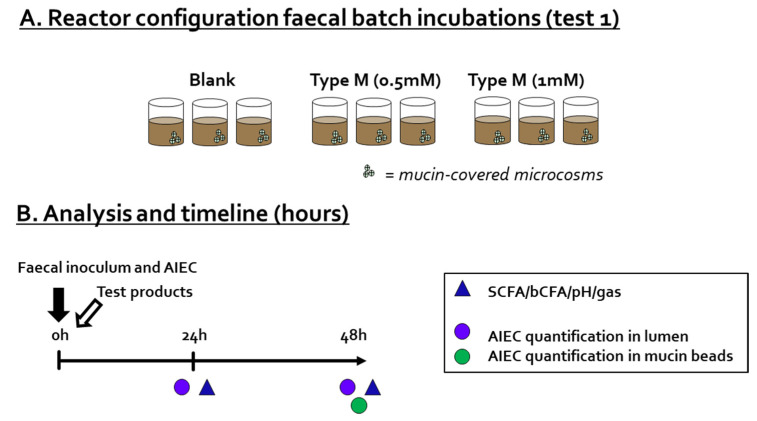
Reactor design (**A**), and timeline and analysis (**B**) of Test 1. The effect of cranberry concentrate Type M (tested at 0.5 mM and 1 mM) was evaluated for the adhesion of AIEC LF82 to mucin-adhered microcosms (*n* = 3).

During Test 1, the impact of a single dose of CTM on AIEC LF82 levels, both in the luminal content and adherence to mucin-covered microcosms, was investigated in the presence of a background microbiota derived from a human adult donor (Figure 1A). Briefly, 49 mL of background medium was added to sterile 100 mL Duran bottles already containing 4 mucin-covered microcosms (AnoxKaldnes K1carrier, AnoxKaldnes AB, Lund, Sweden), prepared according to Van den Abbeele et al. [33]. The reactors also contained 0 mM of CTM (blank), 0.5 mM of CTM, or 1 mM of CTM. Prior to the start of the incubation, 1 mL of overnight-grown AIEC LF82 culture and 20 µL of freshly prepared 7.5% fecal slurry were administered. The fecal slurry was prepared as described by Moens et al. [34]. The dilution of the fecal sample resulted in a microbial dysbiosis, rendering the microbial community vulnerable to AIEC invasion [22]. All reactors were incubated anaerobically at 37 °C for 48 h under continuous agitation (90 rpm), and were run in *n* = 3. Samples were collected at 0, 24, and 48 h for determination of pH, gas production, short-chain fatty acids (SCFA), branched-chain fatty acids (bCFA), and luminal AIEC levels (via qPCR), while mucosal levels of AIEC were determined only at 48 h, since formation of a biofilm requires several days rather than several hours [35].

The AIEC numbers were determined by plating on a selective agar given the presence of a complex background microbiota, i.e., MacConkey (Oxoid Ltd., Basingstoke, UK) containing 50 mg/L of ampicillin and 20 mg/L of erythromycin, allowing the selective enumeration of AIEC LF82 within a complex microbiota [36]. Samples were serially diluted in saline solution (8.5 g/L of NaCl), after which plates were inoculated and incubated aerobically at 37 °C.

### 2.4. Test 2: Adhesion/Invasion and Invasion Assay

#### 2.4.1. Adhesion/Invasion and Invasion Assay 

**Figure 2:**
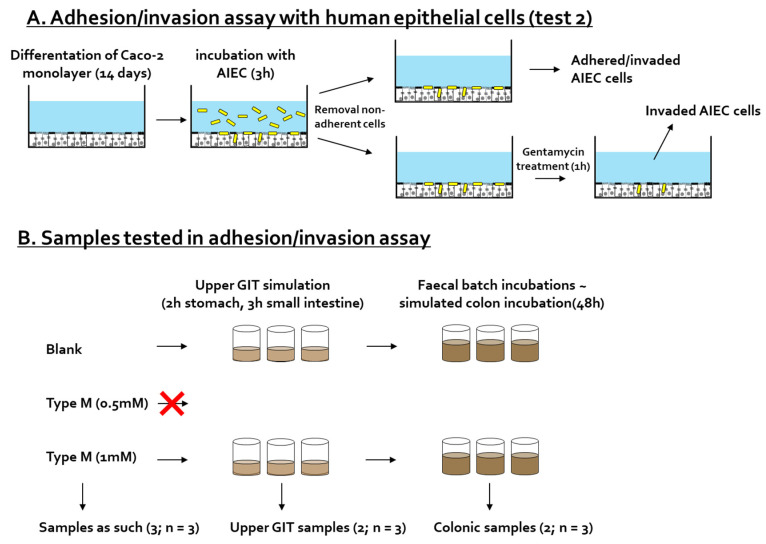
(**A**) Schematic overview of in vitro procedure to test the impact of cranberry concentrate Type M on adhesion/invasion and invasion of AIEC LF82 to human-derived epithelial cells (Test 2). (**B**) Cranberry concentrate Type M was tested as such upon upper GIT incubation and upon simulated colonic incubation (*n* = 3).

For the adhesion/invasion and invasion assay (Figure 2A), Caco-2 cells (HTB-37; American Type Culture Collection) were seeded at 5 × 10^5^ cells in 24-well plates coated with 0.1% gelatin. Cells were grown for 14 days in a complete medium (Dulbecco’s modified eagle medium (DMEM) supplemented with 20% heat-inactivated fetal bovine serum (FBS), 10 mM HEPES, and 1X antibiotic-antimycotic), with 3 medium changes per week. Three days before the experiment, the medium was changed to an antibiotic-antimycotic free complete medium.

The AIEC was cultured for 7 h at 37 °C in nutrient broth, and kept overnight at 4 °C. On the day of the experiment, the cells were incubated in DMEM supplemented with 1% heat-inactivated FBS in the presence or absence of the test products. Bacteria were added to the cells, and after 3 h of incubation, cells were either (i) washed and lysed with 1% triton X-100 (=sum of adhered and invaded AIEC cells) or (ii) further incubated for 1 h in the presence of 250 mg/L of gentamycin, which kills all adhered AIEC cells without disturbing those that invaded the Caco-2 cells (as gentamycin does not permeate within Caco-2 cells). Finally, gentamycin-treated Caco-2 cells were washed and lysed to determine the number of invaded AIEC cells.

In each case, cell lysates and medium controls were plated on nutrient broth agar (non-selective agar, since AIEC was only bacteria present). After 24 h incubation at 37 °C, colonies were counted, and the colony forming units (CFU/mL) were determined.

#### 2.4.2. Preparation of CTM-Containing Samples

The adhesion/invasion of AIEC upon CTM treatment was investigated for (i) the test product as such (at 0.5 mM and 1 mM), (ii) upon simulated digestion of CTM along the upper gastro-intestinal tract (GIT), and (iii) upon subsequent fermentation along the colon (Figure 2B). The CTM was dosed at a concentration of 3.5 mM in the small intestine, so that upon subsequent dilutions, a dose of 1 mM in the colon was reached.

In a fed state, the upper GIT simulation was performed as described by Marzorati et al. [37]. Briefly, the CTM was added to a SHIME nutritional medium, mimicking the stomach. After stomach incubation, pancreatic enzymes and bile salts were added to simulate the small intestinal phase. Incubations were performed at 37 °C under continuous shaking (90 rpm). Prior to being tested on the cells, the small intestinal samples containing 3.5 mM of CTM were diluted to reach a final concentration of 1 mM of CTM.

In addition to the adhesion/invasion assay testing, part of the small intestinal matrix was further incubated in a fecal batch incubation to mimic the colonic fermentation of CTM. The protocol of the fecal batch incubation was similar to the one described above for Test 1, but included slight modifications. Sterile reactors were filled with 43 mL of concentrated background medium (rather than 49 mL of non-concentrated background medium); 20 mL of upper GIT suspension and 7 mL of 7.5% fecal slurry were then added (rather than the 20 µL that was specifically applied to create dysbiosis and AIEC invasion in Test 1). Incubations were performed within a period of 48 h, at 37 °C, under shaking (90 rpm) and anaerobic conditions. In order to account for biological variability, all tests were performed in *n* = 3. Colonic samples collected after 48 h were filtered through a 0.22 µm filter to remove bacteria.

### 2.5. Microbial Metabolic Activity

The acidification during fecal batch incubations is a measure for the overall microbial activity, and was measured using a SenseLine F410 pH electrode (ProSense, Oosterhout, The Netherlands). Furthermore, SCFA (sum of acetate, propionate, and butyrate), bCFA (sum of isobutyrate, isovalerate, and isocaproate), and the sum thereof (total SCFA) were quantified by gas chromatography (GC) coupled with flame ionization detection (FID) as described by De Weirdt et al. [38].

### 2.6. Toxicity Test of Triton X-100 toward AIEC LF82

Prior to the adhesion/invasion assay, the viability of AIEC was evaluated following exposure to the lysis buffer containing triton X-100. Triton X-100 is known to affect the viability of some bacterial species [39]. Therefore, AIEC was cultured for 7 h at 37 °C in nutrient broth and kept overnight at 4 °C. The day of the viability test, AIEC cells were incubated for 30 min in the presence of 1% triton X-100 in PBS. AIEC levels were determined via plating on McConkey agar at the start and at the end of the incubation.

### 2.7. Toxicity Test of CTM, Upper GIT Suspension, and Colonic Suspension toward Caco-2 Cells

Prior to the adhesion/invasion assay, the viability of Caco-2 cells was tested after incubation with upper GIT and colonic suspension. Briefly, Caco-2 cells were again seeded at 5 × 10^5^ cells in 24-well plates coated with 0.1% gelatin. Cells were grown for 14 days in a complete medium (DMEM supplemented with 20% heat-inactivated fetal bovine serum, 10 mM of HEPES, and 1× antibiotic-antimycotic), with 3 medium changes per week. On the day of the toxicity test, cells were incubated with CTM (at 1 mM, 0.5 mM, or 0.25 mM), upper GIT suspension, or colonic suspension. The latter two were diluted in a ratio of 1:5 in a complete medium. After 4 h of incubation at 37 °C, a cell viability assay was performed to assess the toxicity of the compounds, considering the WST-1 assay from Roche Diagnostics GmbH (Germany) that was performed according to the manufacturer’s instructions.

### 2.8. Statistics

To evaluate statistical differences between blank and CTM treatment for each of the endpoints, one-way ANOVA analysis with post hoc Dunnett tests were performed. For metabolic markers, where comparisons were performed on multiple time points, a two-way ANOVA was performed. Significant differences are indicated by (*) = *p* < 0.05 and (**) = *p* < 0.01. Statistical analysis and figure preparation were conducted in GraphPad Prism v9.1.1 software (GraphPad Software, San Diego, CA, USA).

## 3. Results

### 3.1. CTM Stimulated Activity of Background Microbiota and Lowered Colonization of Mucin-Coated Microcosms by AIEC (Test 1)

**Figure 3:**
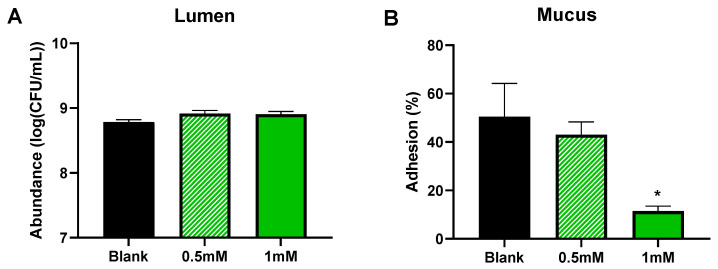
Average (±SEM) AIEC LF82 levels in the lumen (**A**) (log CFU/mL) and mucus (**B**); proportion (%) of the abundance in mucus (CFU/g) versus the abundance in lumen (CFU/mL) during 48 h fecal batch incubations upon treatment with 0 (blank), 0.5, or 1 mM of cranberry concentrate Type M (test 1). Statistical differences versus the blank were calculated using a one-way ANOVA analysis with a post hoc Dunnett test and are indicated with * (*p* < 0.05) (*n* = 3).

At the start of the incubation, 7.43 ± 0.20 log CFU/mL of AIEC LF82 was added to the reactors. AIEC LF82 colonized the reactors up to abundances of around 9 log CFU/mL, with no differences being observed between the blank and CTM treatment in the luminal content (Figure 3A). In contrast, AIEC levels decreased in the simulated mucus layer by 50.5% in the blank to 43.0% and 11.4% when CTM was dosed at 0.5 and 1 mM, respectively (Figure 3B). The decrease was statistically significant following 1 mM of CTM treatment (*p* = 0.032).

**Figure 4:**
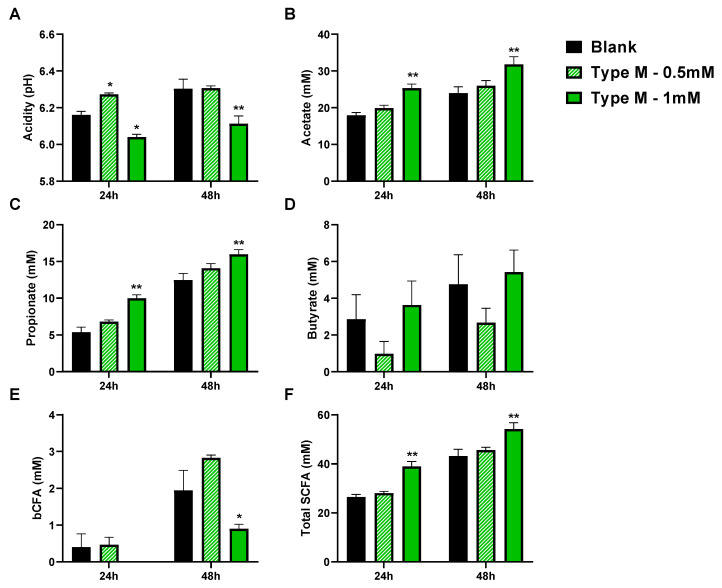
Average (±SEM) acidity (**A**), together with levels of acetate (**B**), propionate (**C**), butyrate (**D**), bCFA (**E**), and total SCFA (**F**) during 48 h fecal batch incubations upon treatment with 0 (blank), 0.5, or 1 mM of cranberry concentrate Type M (test 1). Statistical differences versus the blank were calculated using a two-way ANOVA analysis with a post hoc Dunnett test and are indicated with * (*p* < 0.05) or ** (*p* < 0.01) (*n* = 3).

The lower colonization of the mucus layer by AIEC coincided with an overall more pronounced acidification following incubation with 1 mM of CTM. At 24 h, the pH was lower in the group receiving the dosing of 1 mM (*p* = 0.026), with the effect being even more pronounced at 48 h (*p* = 0.001). This stronger acidification was in part due to the acids present in the test products with an initial pH of 6.65 ± 0.01, 6.57 ± 0.02, and 6.53 ± 0.00 for the blank, and 0.5 and 1 mM of CTM, respectively. This acidification effect could also be the consequence of enhanced microbial metabolism stimulated upon CTM administration (Figure 4), as demonstrated by the increased production of SCFA upon dosing 1 mM of CTM. At 24 h, 1 mM of CTM significantly increased acetate (+7.4 mM; *p* = 0.005), propionate (+4.6 mM; *p* = 0.0004), and total SCFA levels (+12.4 mM; *p* = 0.001). At 48 h, similar effects were observed, with increases in acetate (+7.9 mM; *p* = 0.003), propionate (+3.5 mM; *p* = 0.004), and thus total SCFA levels (+11.0 mM; *p* = 0.003). At 48 h, 1 mM of CTM also decreased bCFA levels (−1.05 mM; *p* = 0.043).

### 3.2. CTM Lowered Adhesion/Invasion and Invasion of Epithelial Cells (Test 2)

**Figure 5:**
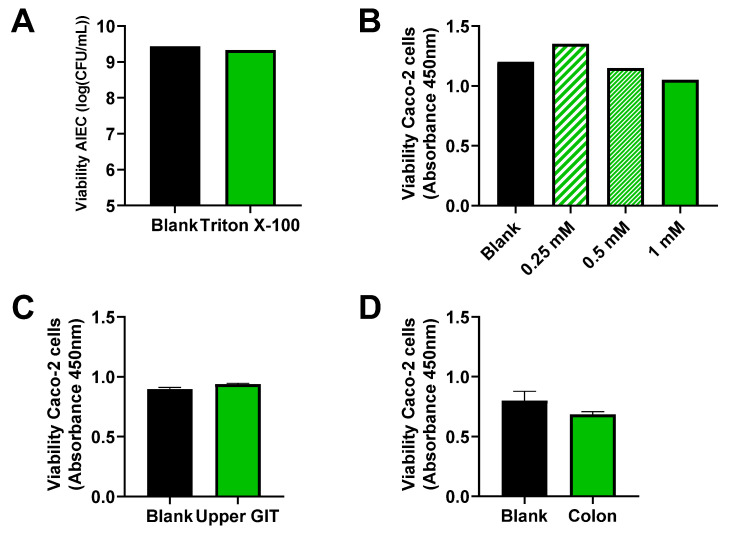
Viability of AIEC LF82 (log CFU/mL) at the end of the lysis procedure with triton X-100 (used to lyse the human-derived epithelial Caco-2 cells during the invasion assay) (*n* = 1) (**A**). Cell viability (as measured via a WST-1 absorbance test) of human-derived epithelial Caco-2 cells following 4 h incubation with CTM (*n* = 1) (**B**), upon undergoing a simulated upper GIT (*n* = 3) (**C**), and colonic incubation (*n* = 3) (**D**), each time versus an untreated blank.

Prior to the adhesion/invasion assay, it was confirmed that AIEC viability was not affected by the lysis procedure that was used to release invaded AIEC cells from the human epithelial Caco-2 cells (Figure 5A).

The toxicity effect of CTM in the upper GIT and colonic matrix on Caco-2 cells was evaluated. In the absence of product and matrix toxicity, one would know that an observed effect is caused by AIEC, but not by the product or matrix. A WST-1 test confirmed that cell viability was not affected by the matrices or by any of the concentrations of CTM tested (Figure 5B–D), so CTM could be tested in the in vitro model under investigation.

**Figure 6:**
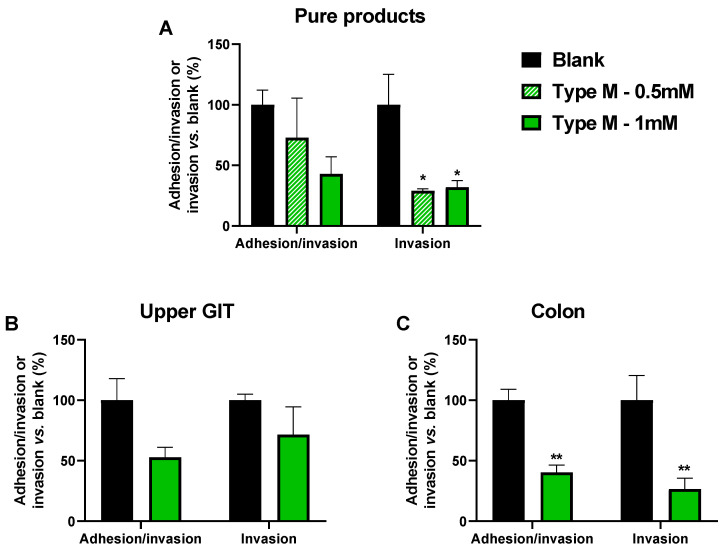
Average (±SEM) AIEC LF82 adhesion/invasion and invasion (expressed as proportion of the viable cells as determined via plating (CFU) in the treatment versus the blank) of human-derived epithelial Caco-2 cells (Test 2). Effects were assessed for the cranberry concentrate Type M (**A**), upon upper GIT incubation (**B**), and upon simulated colonic incubation (**C**). Statistical differences versus the blank were calculated using a one-way ANOVA analysis with a post hoc Dunnett test and are indicated with * (*p* < 0.05) or ** (*p* < 0.01) (*n* = 3).

As in Test 1, the most pronounced effects were observed at the highest test dose of CTM (Figure 6A). While the sum of adhered/invaded AIEC LF82 cells tended to decrease upon dosing 1 mM of CTM (*p* = 0.089), the invaded AIEC LF82 fraction decreased significantly to 32.0% (*p* = 0.043) versus the blank. The 0.5 mM dose did not significantly affect the sum of adhered/invaded cells. However, it significantly decreased the invaded AIEC fraction to 29.1% versus the blank (*p* = 0.035).

Therefore, only the highest dose (1 mM) was tested upon upper GIT (Figure 6B) and colonic incubation (Figure 6C). While upper GIT digestion of CTM seemed to attenuate the effects of CTM on adhesion/invasion, upon colonic fermentation, the effects of CTM were more pronounced. The dose of 1 mM of CTM decreased the sum of adhered/invaded AIEC cells to 40.4% (*p* = 0.009), and the invaded fraction was decreased to 26.6% (*p* = 0.003).

## 4. Discussion

Using a combination of in vitro models ranging from fecal batch incubations to Caco-2 cell adhesion/invasion assays, this in vitro study demonstrated the prebiotic potential of the polyphenol-rich, cranberry-derived product CTM, provided it was dosed in a sufficient amount (1 mM). While prebiotics for dietary applications have often been involved in the use of indigestible carbohydrates such as inulin [40], a recent international scientific consensus suggested including polyphenol-rich products as prebiotics, which are currently defined as a substrate that is selectively utilized by host microorganisms, thus conferring a health benefit [41].

The key health benefit of the CTM was its antipathogenic effect, as demonstrated during the current study using AIEC LF82 as a model organism. Not only did CTM lower the adhesion of AIEC LF82 to mucins, but it also lowered adhesion of AIEC LF82 to human-derived epithelial Caco-2 cells and subsequent invasion by acting on three different levels involved in pathogenic infections [17]. While none of the effects achieved a complete eradication of the pathogen, CTM could exert a potent antipathogenic effect. The effect of cranberry-derived products on *E. coli* is in line with the already reported antimicrobial properties of phenolic compounds extracted from cranberry [28,42,43]. Although anti-adhesive effects have been described previously, such effects were observed in the context of cranberry-derived PACs decreasing P-fimbriae-mediated adhesion of uropathogenic *E. coli* along the urinary tract [25,26]. In contrast to uropathogenic *E. coli*, AIEC LF82 adheres to the cells via type 1 pilli [19], suggesting that the underlying mode of action of the anti-adhesive effect might be different in the gut versus the urinary tract. A potential mechanism worth investigating is the indirect antipathogenic effect via modulation of the background microbiota. Previous studies demonstrated that probiotic strains, such as *Bifidobacterium*, have an anti-adhesion and anti-virulence effect on AIEC in in vitro [44] or inflammatory bowel disease [45]. Further investigation into the stimulation of such beneficial bacteria could complement the direct impact of the CTM on AIEC invasion.

In addition to conferring a health benefit, a second key criterion of the definition of prebiotics is the selective utilization of CTM by the gut microbiota. Such selective utilization is supported by the current study, given the observed selective modulation of metabolic activity with the supplementation of 1 mM of CTM. More specifically, the increase in the acetate and propionate levels following CTM supplementation suggests the selective utilization of CTM by microorganisms that are able to produce these metabolites. An important observation was that CTM decreased the initial pH of the incubation. This provides a potential mode of action by which CTM could impact the gut microbiota. A lower pH favors colonization of acid-resistant *Bifidobacterium* species [46]. Even if CTM is not easily fermented, it might already result in a different outcome of the fermentation by promoting the growth of *Bifidobacterium* species, resulting in an enhanced acetate level [47]. In addition to *Bifidobacterium* species, other microbial species involved in CTM utilization could be *Bacteroidaceae* members. These bacteria were recently found to be selected upon cranberry concentrate administration to an in vitro gut model, at the expense of *Enterobacteriaceae* (to which *E. coli* belongs) [28]. *Bacteroidaceae* are abundant acetate and propionate producers [48]. Another contributing species could be *Akkermansia muciniphila*, another potent acetate and propionate producer [49,50] recently found to be enriched in rodents that were fed a polyphenol-rich cranberry extract, coinciding with protection from diet-induced obesity, insulin resistance, and intestinal inflammation [51]. Independent of the underlying SCFA producing microbes in the current study, both acetate and propionate have been associated with well-documented health benefits. As reviewed by Rivière et al. [52], acetate and propionate both exert anti-inflammatory effects, while propionate specifically promotes satiety, lowers blood cholesterol, decreases liver lipogenesis, and improves insulin sensitivity. Acetate could also serve as an energy source for muscle and brain tissue. Finally, the decrease in bCFA levels upon dosing CTM at 1 mM further suggests a beneficial modulation of the gut microbiota by CTM. bCFA are associated with proteolytic fermentation [53] and the formation of other metabolites with detrimental health effects [54,55]. Altogether, the findings at the metabolic level suggest that CTM could confer health benefits upon its selective utilization by host microorganisms.

Finally, a critical remark on the adhesion/invasion assay needs to be made. If a test product increases or decreases the number of AIEC cells in the medium used to culture the epithelial cells, it will affect the number of cells that can subsequently adhere to or invade the Caco-2 cells. The impact of growth-related effects was minimized in our adhesion and invasion assays with an incubation time as short as 3 h. An additional weakness of the assay is the relatively large variability in the adhesion/invasion and invasion assays. It might warrant more replicates to obtain a statistically robust analysis.

## 5. Conclusions

Overall, the cranberry-derived test product under investigation has prebiotic potential due to the selective stimulation of specific health-related metabolites, and the potent antipathogenic effects against AIEC LF82. Future research should explore the underlying mechanisms and broaden the understanding of the background microbiota during the fermentation of CTM.

## Figures and Tables

**Table 1:** Composition of the cranberry concentrate Type M (CTM). PAC: proanthocyanidin; BL-DMAC: Brunswick Labs 4-dimethylaminocinnamaldehyde method, with procyanidin A2 dimer as standard; OSC DMAC: Ocean Spray Cranberries 4-dimethylaminocinnamaldehyde method, with proprietary PAC extract from cranberries as standard.

Components	Cranberry Concentrate Type M (CTM)
total anthocyanins (mg/kg)	570
organic acids (%)	19.4
sugars (%)	23.4
flavonols (mg/kg)	940
phenolic acids (mg/kg)	1245
PAC--BL DMAC (dwb; mg/kg)	11,250
PAC--OSC DMAC (dwb; mg/kg)	32,975
total phenolics (mg/g)—measured by Folin–Ciocalteu method	15.6
Brix	50.1
